# Genotypic influence in the juvenile to adult transition in olive seedlings

**DOI:** 10.3389/fpls.2024.1343589

**Published:** 2024-02-06

**Authors:** Pedro Valverde, Concepción Munoz Diez, Rustu Efe Deger, Diego Barranco, Carlos Trapero

**Affiliations:** ^1^ Department of Agricultural, Food and Environmental Sciences, Marche Polytechnic University, Ancona, Italy; ^2^ Department of Agronomy (Excellence Unit ‘María de Maeztu’ 2020-23), ETSIAM, University of Córdoba, Córdoba, Spain

**Keywords:** breeding, distance to first flower, field, juvenile period, olive oil, reciprocal crosses, vigor

## Abstract

Olive breeding is a long process and any improvement in shortening the juvenile phase is highly desirable. In the present study, the effect of olive tree parents in different agronomic characteristics have been evaluated during four years in 520 olive genotypes generated from three different crosses in three different experimental fields, all located in Andalusia region, Spain. The crosses evaluated are ‘Arbosana’ x ‘Sikitita’ and its reciprocal, whose parents are characterized by being early bearers; and ‘Frantoio’ free pollinated, whose mother variety is characterized by having a long unproductive period. We studied plant height, distance and time to the first flowering, plant vigor and percentage of olive oil in the fruits. The findings reveal that progeny from ‘Arbosana’ and ‘Sikitita’ crosses, irrespective of the direction of the cross, exhibited a lower distance to flower, early bearing, reduced vigor and a lower percentage of olive oil in fruit compared to ‘Frantoio’ seedlings obtained from free pollination. Furthermore, no discernible differences were observed in the evaluated characteristics when comparing reciprocal crosses across the three fields in the four-years assessment period. Therefore, these results highlight the significance of planting height in reducing the evaluation period required in an olive breeding program and support the hypothesis that there is no maternal effect in the inheritance of the evaluated agronomic characteristics in olive trees.

## 1 Introduction

Olive tree was first domesticated between 6000-8000 years ago in Eastern Mediterranean (Besnard et al., 2013; Diez et al., 2015). Mediterranean Basin hosts more than 95% of the production and genetic variability of the olive tree (Rallo et al., 2018) and for years, producers have selected the most locally suitable cultivars which still reflect the current picture of global olive growing, characterized by a very large number of cultivars with very restricted distribution (Rallo et al., 2018). Despite the large genetic variability still present in the cultivated olive, only a few cultivars have low vigor and adapt to the requirements of the new super high-density olive orchards. Indeed, just few cultivars (‘Arbequina’, ‘Arbosana’, ‘Koroneiki’ and ‘Sikitita’) are being planted in super high-density, and ‘Arbequina’ is massively planted grown super high-density olive orchards worldwide (Centeno et al., 2019). Plant breeding can address this issue by generating new cultivars that combine suitability to high-density growing with other desired characteristics. Therefore, most olive breeding programs focus on increasing the number of cultivars adapted to these conditions and to guarantee the adaptability of new cultivars to emerging olive growing areas and new agronomical threats.

A significant challenge in olive breeding programs is the lengthy duration from the initial crosses to the release of a new variety. This is due to the necessity for olive seedlings to undergo a juvenile and unproductive phase before reaching the adult and productive phase, a process that can extend beyond 14 years, even up to 15 - 20 years (Fontanazza and Baldoni, 1990; Bellini et al., 2002). This characteristic poses a major impediment for breeding programs in olive and other perennials (Rallo, 1995; Rallo et al., 2018).

The development and optimization of techniques to shorten the JP are crucial to increase the efficiency of breeding and to release in a short time new cultivars adapted to new agronomical systems and environmental challenges. Transition in perennials is a progressive phenomenon that entails the presence of a juvenility cone (Hartmann et al., 2002). This is an overall spatial effect within the canopy, in which the upper and peripheral parts of the plant are adult, while the basal and inner parts remain juvenile (Hackett, 1985). As a result, juvenile tissues are localized within the cone-shaped area comprising the trunk and the bases of the lower branches. The existence of the juvenility cone in olive was first observed by Santos-Antunes et al. (2005), corroborated later by Moreno-Alías et al. (2010). The position of the first flower in seedlings has been studied as a marker of the end of the JP, and a juvenile cone has been confirmed in this species (Moreno-Alías et al., 2010; Suárez et al., 2011). The attainment of a certain plant height from the root to the apical meristem is required to flower. In this sense Suárez et al. (2011) reported significative differences in the distance values since root to the first flower among progenies At the same time, correlations with the length of the juvenile period such that early flowering genotypes required a lower minimum distance to the ground to overcome juvenility (De la Rosa et al., 2006).

Plant vigor and genetic inheritance highly affect the JP in olive (Rallo et al., 2008; Hammami et al., 2011; Moral et al., 2011; Moral et al., 2013). The duration of the JP shows an inverse relationship with plant vigor. Accordingly, environmental conditions that reduce vigorous growth, such as mineral deficiency, low light, water stress, defoliation or cold stress, tend to delay the transition from the juvenile to adult phase. In contrast, the conditions that allow for vigorous growth can shorten the period of juvenility (Nocker and Gardiner, 2014). Indeed, the shortening of JP in several fruit species may occur by forcing their growth under optimal conditions (Aldwinckle et al., 1976). This general approach was applied to olive and after years of optimization, the JP was reduced from ~ 15 to 2-4 years by forcing the growth of the seedlings in the greenhouse on continuous lightning, optimal temperature and fertigation (Santos-Antunes et al., 1999). Subsequently, the taller seedlings (> 160 cm) were planted in open fields under drip fertigation and the canopy was trained at 100-130 cm (Moreno-Alías et al., 2010). The duration of the JP in olive trees is linked to a crucial agronomic characteristic for new high-density plantations, known as early bearing (León et al., 2007). Therefore, by choosing plants with a short JP, we are also selecting potential early-bearing cultivars.

Some breeding studies are focused mainly on female genitor due to their so-called dominant heredity. In this sense, although it does not show itself in all areas and conditions, significant female influences were found in tree vigor and length of JP in olive seedlings (Lavee and Avidana, 2011; Moral et al., 2013). Female dominance also varies according to crossing conditions (controlled, self and open) and occurs more profoundly in tree vigor and canopy shape traits (Lavee and Avidana, 2011). Therefore, juvenile to adult transition height might correlate with the female dominance hypothesis. For instance, 50% of the early bearing variety ‘Arbequina’ seedlings flowered an average of 169 cm in length, while the transition occurred in ‘Manzanilla de Sevilla’ seedlings at 211 cm (Moral et al., 2013). Also, Moreno-Alías et al. (2010) determined that in open pollinated ‘Arbequina’ trees, the average minimum length from the trunk base to the first flowers to reach adult status was 200 cm. These results suggest that the transition height depends on the female genitor; therefore, certain critical plant height, a threshold, can be set for different varieties (Moral et al., 2013). However, no further studies have been carried out to confirm this hypothesis.

On the other hand, the possible differential effect of using a cultivar as mother or father in the length of the JP only has been tested using the reciprocal from ‘Manzanilla de Sevilla’ and ‘Arbequina’ crosses and the results suggest no significative differences depending on the direction of the cross (Suárez et al., 2011).

Assessing the existence of this effect and establishing specific flowering distance thresholds for selecting seedlings with short JP depending on their genitors would be crucial to increase the effectiveness of olive breeding programs. To address these two questions, we monitored the vigor, flowering time and flowering height of olive genotypes from different reciprocal crosses at different locations to infer specific flowering patterns and their environmental interactions.

## 2 Materials and methods

### 2.1 Experimental design and plant material

The parental effect on juvenile to adult phase transition and its relation with agronomic characteristics were assessed in three experimental fields. All the trials were located in southern Spain: a) Rabanales Campus from University of Córdoba (UCO) (Córdoba),; b) Pedro Abad (Córdoba) and c) Almensilla (Sevilla) (**Table 1**).

**Table 1:** Climate and soil parameters in the three experimental fields.

	Rabanales (Córdoba)	Pedro Abad (Córdoba)	Almensilla (Sevilla)
Location	37°56’05.5”N	37°57’20.3”N	37°18’51.0”N
	4°42’54.1”W	4°27’45.0”W	6°07’28.7”W
Meters above sea level (m)	151.3	134.2	58.1
Rainfall (mm)*	580.06	558.08	492.35
Average maximum temperature (°C)*	24.42	22.25	24.46
Average medium temperature (°C)*	17.41	17.36	17.51
Average minimum temperature (°C)*	11.02	10.09	11.46
Soil structure	Loam	Clay	Loam-clay

*Climate data for all the experimental field are the average for 23 years (from 01/01/2020 to 31/12/2022).

The evaluated plants belonged to 3 crosses: ‘Arbosana’ x ‘Sikitita’, ‘Sikitita’ x ‘Arbosana’ and ‘Frantoio’ in open pollination. 520 seedlings were generated and evaluated. The parental selection was carried out based on the precocity and importance of the varieties. ‘Arbosana’ and ‘Sikitita’ are some of the few commercially available cultivars adapted to super high-density olive orchards and are characterized by earliness of bearing and low alternate bearing habits (Centeno et al., 2019). ‘Frantoio’ is a well-known Italian cultivar with high vigor and long unproductive period (Tombesi et al., 2011). Directed crosses using ‘Arbosana’ and ‘Sikitita’ olive cultivars were performed in the spring of 2010 and 2013 in trees of the International Olive Germplasm Bank of Cordoba (UCO Collection) according to a UCO optimized protocol by applying pollen (collect from the trees of the variety that will act as father) to bagged branches with flowers (in the tree that will be the mother in the directed crossing) (Valverde et al., 2021). Seed extraction and classification after removing the fruit flesh and pit were done in autumn after the harvest and seeds were disinfected with Tiram 50% and placed in Petri dishes containing wet perlite kept at 14°C for 30 days. A forced-growth protocol was followed after germination (Santos-Antunes et al., 2005). Seedlings were transplanted into 2.5 L pots and transferred to the greenhouse where drip fertigation, controlled temperature (24°C on average) and continuous lightning for 24 hours were provided. Seedlings were planted in the three fields with different heights (50-125 cm) and 4 m x 1.5 m spacing distributed in 4 blocks in each field (**Table 2**). All the experimental fields were cultivated following the standard practice of commercial orchards: annual irrigation was 2000 m^3^/ha applied by drip irrigation. Soil management included herbicide application under the trees and spontaneous vegetative cover between rows, controlled by soil cultivation. Pesticides and fungicides were applied when necessary, following a integrated pest and disease management principles.

**Table 2:** Number of seedlings from the different crosses planted in each experimental field.

Experimental	Year of seed	Plantation	Female	Male	Number of
field	germination	year	genitor	genitor	genotypes
Pedro Abad			‘Arbosana’	‘Sikitita’	204
(Córdoba)	2011	2012	‘Sikitita’	‘Arbosana’	–
			‘Frantoio’	Open pol.	96
Rabanales			‘Arbosana’	‘Sikitita’	30
(Córdoba)	2013	2014	‘Sikitita’	‘Arbosana’	30
			‘Frantoio’	Open pol.	24
Almensilla			‘Arbosana’	‘Sikitita’	58
(Sevilla)	2014	2015	‘Sikitita’	‘Arbosana’	59
			‘Frantoio’	Open pol.	19

### 2.2 Genotype evaluation

The trials were set up and evaluated in different years in each field according to the plantation year (**Table 2**). Seedlings were planted into the field in 2012, 2014 and 2015 in Pedro Abad (Córdoba province), Rabanales (Córdoba province) and Almensilla (Sevilla province), respectively. Evaluations were performed continuously for four years with an additional 5th year in Pedro Abad and Rabanales. Ontogeny of the plants (juvenile/adult stage), plant vigor and productivity and olive oil percentage were recorded as described:

#### 2.2.1 Juvenile to adult transition

The number of flowering genotypes were assessed each May. First flowering distance was measured by adapting the method proposed by Moreno-Alías et al. (2010). The branches with flowers were identified and their distance to the ground was measured in 3 branches. The measurement was made first by taking the height of the trunk until the main branch on which the flower was found and then measuring the sub-branches until reaching the location of the flower (Trunk distance + Branch*1* distance + Branch*2* distance + Branch*n* distance = D*fl*). The shortest distance from the ground to flower was selected for each genotype for the analysis. The time needed for JP to adult transition was given as monthly difference since germination.

#### 2.2.2 Plant vigor

The planting height of the seedlings were taken along with the trunk diameter. Measurements were repeated once a year in the winter season to test the parental effect on vigor.

#### 2.2.3 Olive oil percentage

In Rabanales experimental field, approximately 300 g of fruit were sampled in the version stage to assess oil quantity (Pardo et al., 2021). The sampling was carry out during 2016 to 2019 years in middle of November and all those plants that had fruit were sampled. The oil percentage was measured by an NMR analyzer Minispec NMS100 (Bruker Optik GmbH, Ettlingen, Germany) according to the protocol developed by Del Río and Romero (1999).

### 2.3 Statistical analysis

All the statistical analyses have been performed using the Statistix 10.0 software (Analytical Software, Tallahassee, Fl, USA). The following parameters were performed based on randomized block design with replications in each block: Time to first flower, genotypes in flowering by years, first flower distance, plant height year of first flower, plant height, trunk diameter and olive oil content. In all parameters, ANOVA was performed. Variance (ANOVA) analysis was applied and significant differences among means were compared using Fisher’s protected least significant difference (LSD) test (α = 0.05).

## 3 Results

### 3.1 Parental effect in the juvenile to adult transition

**Figure 1:**
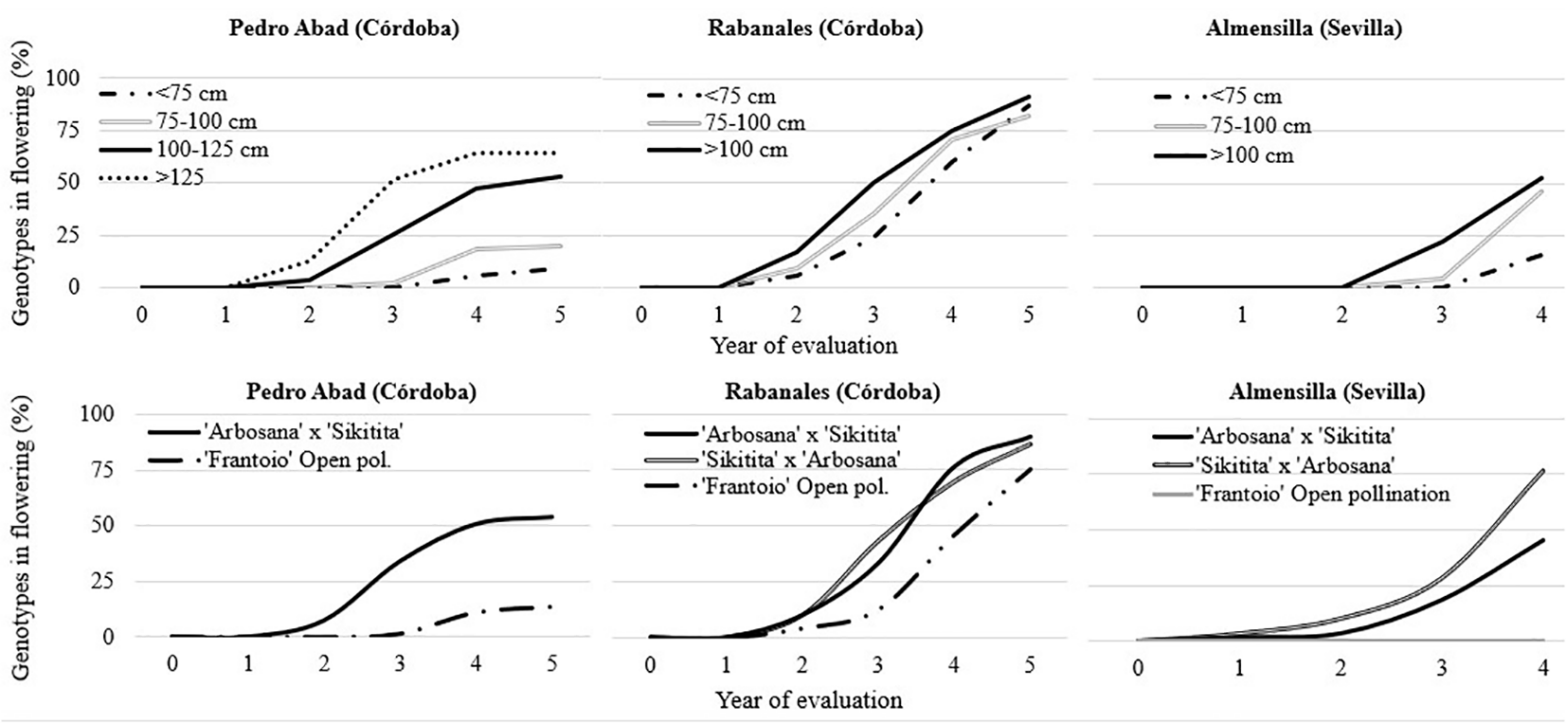
Percentage of genotypes in flowering during the years of evaluation in the three experimental fields depending on the planting height and crosses. The year 0 represents the plantation day.

The first flowers were observed in ‘Arbosana’ x ‘Sikitita’ and its reciprocal cross one year after plantation in Almensilla. The second year after planting, genotypes from the reciprocal cross started to flower in all the experimental fields with no significative differences. Conversely, the seedlings of ‘Frantoio’ in open pollination only had genotypes with flowering in the Rabanales field after two years, with a significantly lower rate (**Table 3**). The time needed for the juvenile to adult transition phase in ‘Frantoio’ open pollination was mostly higher and progressed at a lower rate over time. Compared to other experimental fields, a significant portion of the trees in Rabanales reached the adult stage in 4 years, with 64.3% on average (**Figure 1**). There were no significative differences between reciprocal crosses with 77 and 70% of ‘Arbosana’ x ‘Sikitita’ and ‘Sikitita’ x ‘Arbosana’ seedlings with flowering (adult phase) after four years, whereas 45.8% of ‘Frantoio’ seedlings flowered in the same period. Significantly less flowering was observed in Pedro Abad and Almensilla than in Rabanales, 31% and 40.6%, respectively. The only exception was ‘Sikitita’ x ‘Arbosana’ in Almensilla, with 76.7% flowering in the same period. ‘Frantoio’ had the lowest number of adult seedlings in all experimental fields. No flowering was observed in ‘Frantoio’ open pollination in Almensilla, while in Pedro Abad it was only 11.1%. During all the years of evaluation, the percentage of flowering genotypes from the reciprocal cross ‘Arbosana’ x ‘Sikitita’ and ‘Sikitita’ x ‘Arbosana’ was higher than that obtained in the offspring of ‘Frantoio’ in open pollination in the three fields evaluated (**Table 3**). Regarding the planting height, in general the same result was obtained in the three experimental fields. The genotypes planted with less than 75 cm reached the adult phase, that is, they had flowering, later than the genotypes that had been planted with a greater height. The genotypes with more than 125 cm get the adult phase before than the rest of the genotypes (**Figure 1**). In the case of Pedro Abad and Almensilla, less than 20% of the genotypes that were planted with less than 75 cm flowered in 4 years of experiment. On the other hand, in Rabanales they reached 70% of genotypes flowering in 4 years (**Figure 1**).

**Table 3:** Genotypes flowering during the 6 years of evaluation in the three experimental field.

Cross	Experimental	Plants	Genotypes in flowering by year (%)					
	field	n°	0*	1	2	3	4	5
‘Arbosana’ x ‘Sikitita’	Pedro Abad	204	0	0.0 a	7.4 a	34.1 a	51.0 a	54.1 a
‘Sikitita’ x ‘Arbosana’		0	–	–	–	–	–	–
‘Frantoio open pollination		96	0	0.0 a	0.0 b	1.5 b	11.1 b	13.6 b
**Average**		**300**	**0**	**0**	**3.7**	**17.8**	**31**	**33.9**
‘Arbosana’ x ‘Sikitita’	Rabanales	30	0	0.0 a	10.0 a	33.0 a	77.0 a	90.0 a
‘Sikitita’ x ‘Arbosana’		30	0	0.0 a	10.0 a	43.0 a	70.0 a	87.0 a
‘Frantoio open pollination		24	0	0.0 a	4.2 b	12.5 b	45.8 b	75.0 b
**Average**		**84**	**0**	**0**	**8.1**	**29.5**	**64.3**	**84**
‘Arbosana’ x ‘Sikitita’	Almensilla	58	0	1.7 a	3.3 ab	18.3 a	45.0 ab	–
‘Sikitita’ x ‘Arbosana’		59	0	3.3 a	10.0 b	28.3 a	76.7 a	–
‘Frantoio open pollination		19	0	0.0 b	0.0 b	0.0 b	0.0 b	–
**Average**		**136**	**0**	**1.7**	**4.4**	**15.6**	**40.6**	

*Plantation year. The comparison has been made between crosses of each field, that is, the three fields have not been compared at the same time. Values in columns followed by the same letter are not significantly different at a probability level of α = 0.05 according to the Fisher’s protected least significant difference (LSD) test.

### 3.2 First flowering distance and plant vigor

**Figure 2:**
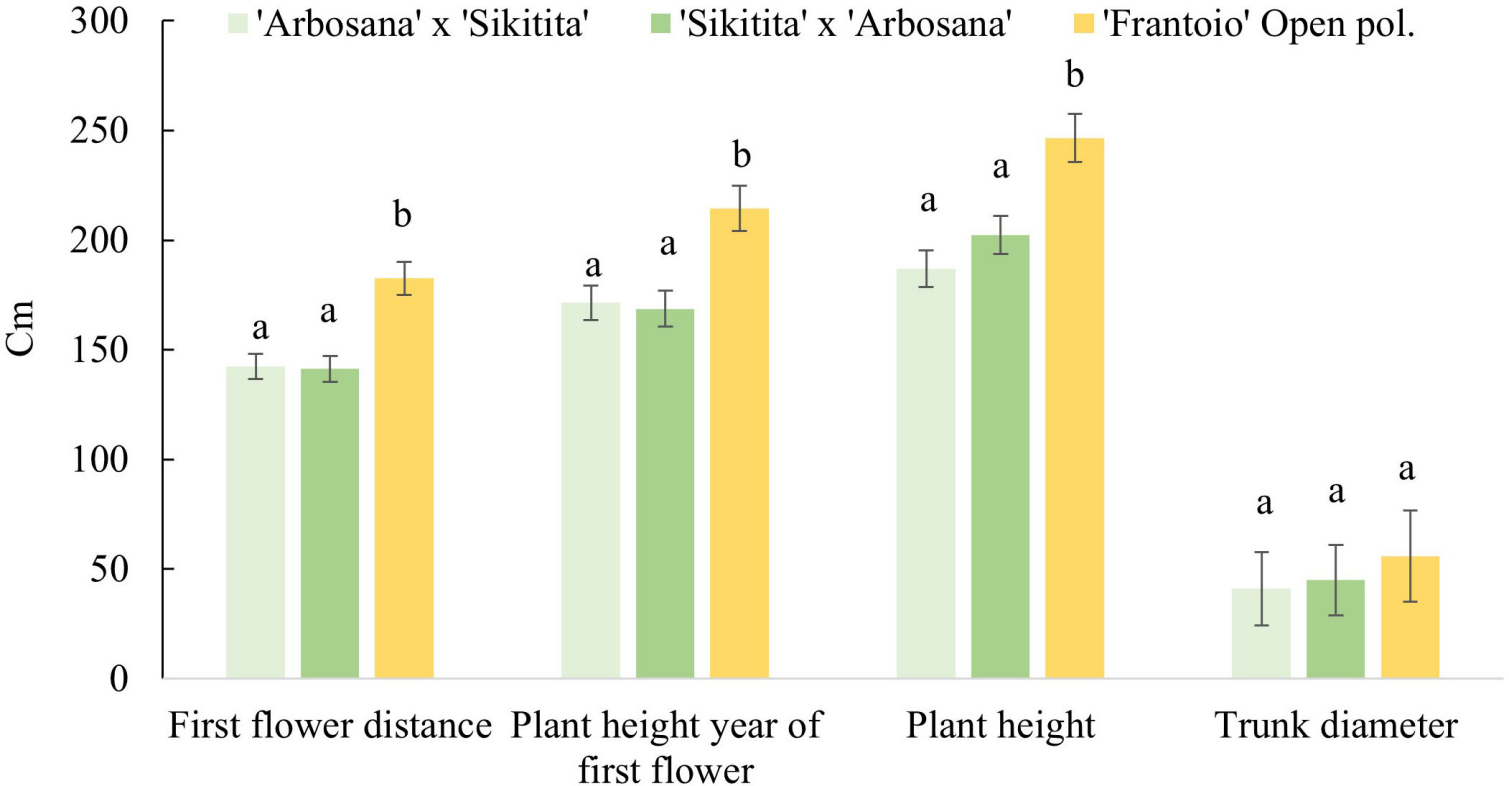
Distance since trunk base to first flower, height of the plants the year of the first flower, plant height and trunk diameter the last year of evaluation in the genotypes evaluated in the experimental field “Rabanales”.

Reciprocal crosses differed from ‘Frantoio’ seedlings with a lower flowering distance with average values in Pedro Abad (266 cm), Rabanales (162 and 174 cm) and Almensilla (172 and 177 cm) against the obtained by ‘Frantoio’ with 294 cm in PedroAbad and 211 cm in Rabanales. The difference between ‘Arbosana’ x ‘Sikitita’ and ‘Sikitita’ x ‘Arbosana’ crosses was no significative (**Table 4**). Similarly, the plant height was always higher than the other crosses in ‘Frantoio’ seedlings. Regarding vigor, plant height and trunk diameter values have also been significantly higher in the offspring of the ‘Frantoio’ than in the rest of the crosses. In this sense, no significative parental effect was found when we compared the first flowering distances and vigor parameters (height and trunk diameter) in the reciprocal crosses in experimental sites and duration (**Figure 2**). Also, no significant differences were found among fields first flowering distance.

**Table 4:** Minimun, maximun, average first flower distance and average plant height in the three experimental field.

Experimental	Crosses	Minimum	Maximum	Average	Average
field		distance (cm)	distance (cm)	distance^a^ (cm)	Plant height^b^ (cm)
Pedro Abad	‘Arbosana’ x ‘Sikitita’	90	330	190a	266a
	‘Sikitita’ x ‘Arbosana’	–	–	–	–
	‘Frantoio’ Open Pol.	130	420	234b	294b
Rabanales	‘Arbosana’ x ‘Sikitita’	85	209	141a	162a
	‘Sikitita’ x ‘Arbosana’	85	273	138a	174a
	‘Frantoio’ Open Pol.	90	275	161b	211b
Almensilla	‘Arbosana’ x Sikitita	102	192	150a	172a
	‘Sikitita’ x ‘Arbosana’	104	203	155a	167a
	‘Frantoio’ Open Pol.	–	–	–	–

a Values in columns followed by the same letter are not significantly different at a probability level of α = 0.05 according to the Fisher’s protected least significant difference (LSD) test.

b Average plant height the same year of distance evaluation.

### 3.3 Olive oil content

**Figure 3:**
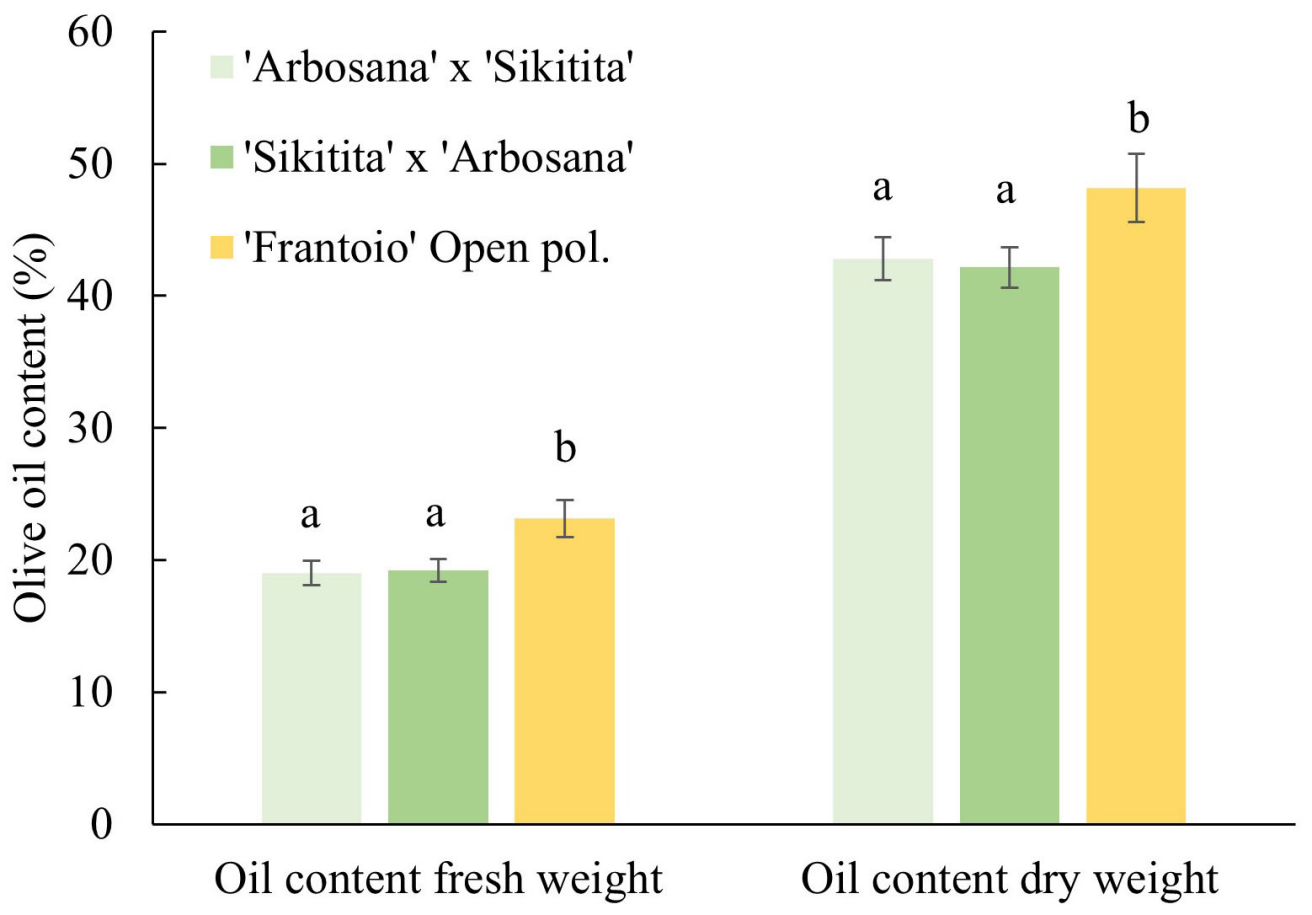
Comparison of olive oil content in the three crosses in the Rabanales experimental field.

In parallel with the parameters evaluated previously, there were no significant differences in olive oil contents, both in fresh and dry weight, between ‘Arbosana’ x ‘Sikitita’ and ‘Sikitita’ x ‘Arbosana’ crosses, with average values of oil 19.0% and 19.2% fresh and 42.8% and 42.1% dry weight, respectively. On the other hand, the main differences were obtained when we compared the reciprocals with ‘Frantoio’ open pollination. Oil content was 23.1% and 48.2% fresh and dry weight on average (**Figure 3**).

## 4 Discussion

Modern agriculture faces ongoing important challenges such as pests or diseases. The development of new varieties is essential not only to address existing problems but also to provide new characteristics demanded by consumers. Unlike annual crops, where breeding programs continually produce new varieties cutting-edge technologies and molecular breeding (Fujino et al., 2017; Guzmán et al., 2019; Cappetta et al., 2020; Merrick et al., 2022), most perennial fruit crops present a different scenario. While certain crops, such as peach (Nardino et al., 2022), plum (Castro et al., 2013) or apple (Teh et al., 2021), continually develop and implement new varieties, other crops such as pistachio (Sheikhi et al., 2019) or olive tree (Rallo et al., 2018) show a slower adoption of new varieties, mainly due to the deep-rooted use of conventional varieties (Marchese et al., 2016). Additionally, the long transition from the juvenile to the adult phase further extends the timeline for developing new varieties (Vargas et al., 2002; Rallo et al., 2018).

Specifically, in olive growing, a breeding program may take more than 14 years due to the long juvenility, and evaluation of agronomic traits such as productivity or olive oil quality can begin once the adult phase has passed (Rallo et al., 2018). Even in some cases, the unproductive period of the olive tree ranges from 15 to 20 years (Fontanazza and Baldoni, 1990). However, later studies demonstrate that an accurate selection of genitors and only planting genotypes with specific characteristics in the field can significantly reduce this unproductive period to less than 5 years (De la Rosa et al., 2006; Rallo et al., 2008; Moral et al., 2013; Valverde et al., 2023).

This study has evaluated the necessary time for pass from juvenile to adult in olive seedling during at least 4 years in three different fields in the south of Spain, exploring how the first flowering is affected by three olive progenies derived from parents with a short and long unproductive period and planting heights. Additionally, it has been evaluated the potential maternal or paternal influence on the inheritance of traits like vigor and oil content, comparing the reciprocal crosses ‘Sikitita’ x ‘Arbosana’ and ‘Arbosana’ x ‘Sikitita’ and the seedling from ‘Frantoio’ open pollinated.

As in previous works, it is corroborated that the short (‘Arbosana’ and ‘Sikitita’) or long (‘Frantoio’) unproductive period of the parents used in the crosses translates into a short or long JP of their seedlings (De la Rosa et al., 2006; León et al., 2007). As a result of planting seedlings shorter than 75 cm in the orchards, juvenile period was extended in the reciprocal crosses from ‘Arbosana’ and ‘Sikitita’. Still, some genotypes managed to produce flowers in the evaluation period, regardless of the planting height, due to the short unproductive time of the parents. ‘Frantoio’ is characterized by an extended juvenile period and high vigor. Our study reflects these traits, as there were no flowering seedlings in Almensilla over four years, despite variations in planting heights. It also seems possible to find an effective threshold to reduce juvenile phase of this cultivar when planting heights are increased in different varieties (Moral et al., 2013). Conversely, Moral et al. (2013) concluded that cultivar-specific height thresholds should be determined by considering the mother plant, Our results align partially with those obtained by Moral et al. (2013), where crosses made in 1998 and planted with less than 75 cm did not produce flowers after 5 years of evaluation due to the prolonged non-productive period of the parent plants, particularly evident in the case of ‘Frantoio’ in open pollination. This pattern was observed in our study in the Pedro Abad and Almensilla experimental fields with average values indicating that fewer than 20% of genotypes displayed flowering within the 4-year evaluation period.

The influence of planting height on juvenile to adult phase correlates with the vigor of the specific olive variety. In our study, a planting height of 100 cm was found effective in reducing juvenile period to 1 year in varieties with a low juvenile period such as ‘Arbosana’ and ‘Sikitita’ similarly to previous works (De la Rosa et al., 2006; León et al., 2007). Furthermore, from the second year after planting, flowering was observed in all crosses and experimental fields, although the percentages of genotypes flowering were growing progressively, except for open pollinated ‘Frantoio’ in Almensilla, whose genotypes did not flower during 4 years of evaluation. This observation highly contrasts with the 15 to 20 years necessary to reach the adult phase reported by Fontanazza and Baldoni in 1990, although progenies with a long unproductive period were evaluated in that case.

Certain genotypes flowered at around 100 centimeters, indicating the early bearing characteristics to each genotype. In this work, the mean distance of the first flowering has been evaluated and the values obtained have varied from 138 cm for ‘Sikitita’ x ‘Arbosana’ in Rabanales to 234 cm in the offsprings of ‘Frantoio’ in open pollination in Pedro Abad. Distance to first flower also varied between different experimental fields, for instance, ‘Arbosana’ x ‘Sikitita’, the distances were 141, 150, and 191 cm in Rabanales, Almensilla and Pedro Abad, respectively. Moreno-Alías et al. (2010) determined that 200 cm is the average distance for ‘Arbequina’ open pollination crosses, with values ranging from 157 cm to 260 cm. Approximately 100 cm variation is due to an unknown pollen source but also indicates parental effect. These values agree with those obtained in the present work with slight exceptions in data due to the different environmental conditions (Moreno-Alías et al., 2010; Suárez et al., 2011). Moreover, there were no significant different in the first flower distance across the 3 experimental sites, suggesting that this trait is mostly genetically influenced when given a similar management but different locations. The first flower distances were not significant among reciprocal crosses of ‘Arbosana’ and ‘Sikitita’, which corroborates with the results of Suárez et al. (2011) where no significative differences were found in the distance of the first flower of the reciprocal crosses from ‘Manzanilla de Sevilla’ and ‘Arbequina’ with the values 234 and 243 cm, respectively. Our results support the idea that both the mother and the father influence this trait equally and are equally important in selecting parents in breeding programs.

The non-significant differences in oil contents of reciprocal crosses also support the equal contribution of mother and father genitors. Furthermore, no specific maternal influence was found in the breeding study conducted by Valverde et al. (2021) with five different reciprocal hybrids against Verticillium wilt caused by *Verticillium dahliae*. Similar results have been previously reported in other crops, such as almond, blossom density, percentage of double kernels and kernel smoothness was not affected by reciprocal crosses. However, parental effect is still effective in some characteristics, such as kernel ratio or time of ripening, and suggests that the direction of crossing should be considered for the targeted parameters (Dicenta and Garcia, 1993). Although no parental effect was observed in various characteristics of olive tree investigated in this study, likely because these traits are under a polygenic control, thus in F1 progenies is not possible to detect their segregation, further research is needed to determine their influence on other important traits such as disease and pest resistance and olive oil quality parameters.

We have demonstrated that first flower distance in olive is predominantly influenced by genetic factors, making it a stable and comparable selection trait in breeding programs, provided an adequate farm management. This trait holds significant importance in the selection of early-bearing genotypes, which is currently a key trait not only in olive growing but also in the context of any perennial crop.

## Data availability statement

The raw data supporting the conclusions of this article will be made available by the authors, without undue reservation.

## Author contributions

PV: Conceptualization, Data curation, Formal analysis, Funding acquisition, Investigation, Methodology, Project administration, Resources, Supervision, Validation, Visualization, Writing – original draft, Writing – review & editing. CM: Conceptualization, Investigation, Methodology, Supervision, Validation, Visualization, Writing – review & editing. RD: Data curation, Software, Visualization, Writing – original draft. DB: Conceptualization, Funding acquisition, Project administration, Resources, Visualization, Writing – review & editing. CT: Conceptualization, Data curation, Formal analysis, Funding acquisition, Investigation, Methodology, Project administration, Resources, Supervision, Validation, Visualization, Writing – original draft, Writing – review & editing.
